# Circulating IgM Requires Plasma Membrane Disruption to Bind Apoptotic and Non-Apoptotic Nucleated Cells and Erythrocytes

**DOI:** 10.1371/journal.pone.0131849

**Published:** 2015-06-29

**Authors:** Emily E. Hesketh, Ian Dransfield, David C. Kluth, Jeremy Hughes

**Affiliations:** MRC Centre for Inflammation Research, Queen’s Medical Research Institute, University of Edinburgh, Edinburgh, Scotland; Medical College of Georgia, UNITED STATES

## Abstract

Autoimmunity is associated with defective phagocytic clearance of apoptotic cells. IgM deficient mice exhibit an autoimmune phenotype consistent with a role for circulating IgM antibodies in apoptotic cell clearance. We have extensively characterised IgM binding to non-apoptotic and apoptotic mouse thymocytes and human Jurkat cells using flow cytometry, confocal imaging and electron microscopy. We demonstrate strong specific IgM binding to a subset of Annexin-V (AnnV)^+^PI (Propidium Iodide)^+^ apoptotic cells with disrupted cell membranes. Electron microscopy studies indicated that IgM^+^AnnV^+^PI^+^ apoptotic cells exhibited morphologically advanced apoptosis with marked plasma membrane disruption compared to IgM^-^AnnV^+^PI^+^ apoptotic cells, suggesting that access to intracellular epitopes is required for IgM to bind. Strong and comparable binding of IgM to permeabilised non-apoptotic and apoptotic cells suggests that IgM bound epitopes are 'apoptosis independent' such that IgM may bind any cell with profound disruption of cell plasma membrane integrity. In addition, permeabilised erythrocytes exhibited significant IgM binding thus supporting the importance of cell membrane epitopes. These data suggest that IgM may recognize and tag damaged nucleated cells or erythrocytes that exhibit significant cell membrane disruption. The role of IgM *in vivo* in conditions characterized by severe cell damage such as ischemic injury, sepsis and thrombotic microangiopathies merits further exploration.

## Introduction

The pathophysiology of many autoimmune diseases is associated with perturbations in the highly regulated processes of apoptosis and subsequent clearance of apoptotic cells by phagocytes [1–3]. During apoptosis, sequential activation of initiator/effector caspases leads to morphological changes including cell shrinkage and nuclear pyknosis, with maintenance of plasma membrane integrity [4–6]. In the absence of efficient phagocytic clearance, apoptotic cells ultimately progress to secondary necrosis, with the loss of membrane integrity and release of potentially immunogenic intracellular contents, including organelles [7]. In contrast, high levels of necrotic death is found in some diseases, following critical cell damage resulting from hypoxia, toxins or infection. Cells undergoing primary necrosis exhibit catastrophic loss of membrane integrity and rapid release of potentially pro-inflammatory intracellular components [8, 9]. Such a situation occurs in patients with thrombotic microangiopathy syndromes [10] in which microvascular thrombus formation and enhanced shear stress [11, 12] disrupt and fragment erythrocytes [12–14]. Erythrocyte fragmentation with the release of cytotoxic cell-free haemoglobin and damage associated molecular pattern (DAMP) exposure would be predicted to further drive inflammation [15–17]. It is therefore critical that apoptotic cells, necrotic cells and severely damaged erythrocytes are rapidly removed in order to limit potential autoimmunity and tissue and vascular inflammation.

The development of autoantibodies, particularly against cytoplasmic or nuclear antigens which characterizes diseases such as systemic lupus erythematosus (SLE), has been suggested to be a consequence of excessive levels of apoptosis and/or compromised clearance [18]. However, the immunogenicity of dying cells is not simply determined by the mode of cell death (apoptosis versus necrosis), suggesting that there are additional molecular determinants that influence autoantibody generation. The repertoire of membrane alterations associated with cell death [19], including exposure of phosphatidylserine (PtdSer) [20] on the outer plasma membrane leaflet together with binding of specific opsonins or bridging molecules, such as C-reactive protein, Protein S, milk fat globule-EGF factor 8, C1q, mannose-binding lectin or IgM antibodies [21–23] determine the molecular pathways utilized in phagocytic uptake. Furthermore, the opsonization status of apoptotic or necrotic cells will govern the ultimate fate of internalized cellular material within phagocytes and the capacity for presentation of antigens to T cells that is necessary for B cell activation and autoantibody production [8].

Immunoglobulin class M (IgM) antibodies have been shown to mediate and accelerate the clearance of apoptotic cells by phagocytes [24–27], a function that may involve recruitment of C1q [28, 29]. Phagocytosis of apoptotic cells is markedly reduced in the absence of IgM [30] and IgM-deficient mice exhibit a clear autoimmune phenotype similar to that seen in SLE [31, 32]. Circulating IgM antibodies are of relatively low affinity and exhibit polyreactivity [33–35] as they bind to a range of non-self and self-antigens [36–39]. These IgM bound antigens are diverse and include carbohydrate antigens, such as chitin and cell wall polysaccharides [40, 41], cytoskeletal proteins, for instance actin, non-muscle myosin heavy chain II, myosin and B-tubulin [36, 38, 42] as well as nuclear antigens [43–46]. Late apoptotic cells [26, 36, 47–49] and derived microparticles [27] are preferentially bound by IgM antibodies. Consequently, IgM antibodies have been reported to bind neo-epitopes that become accessible following plasma membrane phospholipid alterations associated with apoptosis [36, 50, 51], such as exposure of phosphorylcholine [52] and PtdSer [27, 36]. In addition, membrane phospholipase (PLA2) has been implicated in phospholipid remodeling and exposure of IgM bound epitopes [53]. Many IgM antibodies recognise oxidation-specific epitopes that are present on apoptotic cells and oxidized low-density lipoproteins (LDL) [41, 54–56], such as LDL-malondialdehyde [41, 57, 58] and lysophosphatidylcholine [51, 59], representing immunodominant DAMPs [60, 61]. Whilst the precise molecular determinants on apoptotic cells that are recognized by IgM are yet to be fully defined, an important role for IgM in the clearance of apoptotic cells is well established [24–27, 31, 32].

In this paper, we sought to explore why circulating IgM antibodies may exhibit preferential binding to late apoptotic cells. As membrane integrity differs considerably between early and late apoptotic cells [4–7] we hypothesised that the degree of membrane disruption may influence accessibility of IgM binding such that IgM may bind intracellular epitopes. We carefully characterized IgM binding to cells undergoing apoptosis and demonstrate robust binding of both human and murine IgM to a subset of cells that have exposed PtdSer (Annexin V (AnnV) positive) and exhibit loss of membrane integrity (Propidium Iodide (PI) positive). Confocal and electron microscopy studies indicate that IgM binds to cells at a morphologically advanced stage of apoptosis with evident disruption of the plasma membrane. IgM binding thus requires loss of plasma membrane integrity allowing access to cytoplasmic or cell membrane antigens. Importantly, we show that IgM strongly binds non-apoptotic cells and erythrocytes that have undergone membrane permeabilisation suggesting that the epitopes bound are present within normal cells and not necessarily generated by the apoptotic process *per se*. These findings suggest an important role for IgM in the recognition of nucleated cells and erythrocytes that have lost significant membrane integrity.

## Materials and Methods

### Mice

Balb/c or RAG1-deficient mice were purchased from Harlan, UK or Charles River. Mice were housed in the Biomedical Research Resource Unit at the University of Edinburgh and were given *ad libitum* access to food and water. Animal procedures were performed in accordance with guidelines set out by the United Kingdom’s Home Office under the Animal (Scientific Procedures) Act of 1986 and were approved by the University of Edinburgh’s Biological Services Department. Mice were sacrificed by cervical dislocation according to the United Kingdom’s Home Office Scientific Procedures Act Schedule 1 regulations. Total mice used: 99.

### Chemicals and cell culture reagents

Cell culture reagents were purchased from Life Technologies, Paisley, UK. Chemicals and reagents were purchased from Sigma Aldrich, UK unless stated otherwise.

### Preparation of non-apoptotic and apoptotic thymocytes

Following cervical dislocation thymi were dissected from 4–8 week old male Balb/c mice. Thymocytes were dissociated by grinding thymi through a cell strainer (40μM nylon, BD Falcon). Thymocytes were used immediately or aged overnight in RPMI 1640 at 37°C and 5% CO_2_ to generate a cell population with variable levels of apoptosis. Following incubation cells were centrifuged at 230 x *g* for 6-minutes, and washed with PBS.

### Culture of Jurkat cells and induction of apoptosis

Human Jurkat cells [62] were cultured in RPMI 1640 with 10% heat-inactivated fetal calf serum (FCS), L-glutamine and penicillin-streptomycin at 37°C and 5% CO_2_. Cells were cultured at a density of 2x10^6^/mL and apoptosis induced by incubation with 0.5μM camptothecin for 16-hours. Cells were then centrifuged at 300 x *g* for 6-minutes, and washed with PBS.

### Culture of human proximal tubular epithelial cells (HK-2)

HK-2 cells [63] (kindly provided by Dr Mark Dockrell (SWT Intitute for Renal Research, London)) were cultured in RPMI 1640 with 5% heat-inactivated FCS, L-glutamine and penicillin-streptomycin at 37°C and 5% CO_2_. HK-2 cells were detached with trypsin or cell dissociation buffer. Morphology was assessed by microscopy using a Zeiss Axiovert S100 with an x32/0.4 objective and imaged using a CoolSnap RS Photometrics camera and OpenLab software. Whole image contrast/brightness and pseudocolour was adjusted using ImageJ (NIH).

### Permeabilisation

Cells (excluding erythrocytes) were permeabilised using the following saponin based reagents purchased from eBioscience and used according to manufactures instructions: Fixation/Permeabilisation Concentrate (Ref: 00-5123-43), Fixation/Permeabilisation Diluent (Ref: 00-5223-56) and Permeabilisation Buffer 10x (Ref: 00-8333-56). To confirm permeability 1μL PI [1mg/mL] (Invitrogen, UK) diluted 1:20 in PBS was added to samples before analysis by flow cytometry on a BD FACSCalibur. For confocal microscopy permeability was confirmed by Annexin-V staining only as described in ‘Annexin-V (AnnV) and Propidium Iodide (PI) staining’.

### Assessment of IgM binding

Approximately 5x10^5^ cells (excluding erythrocytes) were incubated in PBS or 30% human serum (Sigma Aldrich) (Jurkat and HK-2) or 12.5% Balb/c or RAG1-deficient mouse plasma (thymocytes) for 30-minutes. For thymocytes APC Mouse (Balb/c) IgM κ isotype control (25μg/mL, Clone: G155-228, BD Pharmingen) was used as control irrelevant mouse IgM antibodies to assess non-specific interaction. This was used at a high concentration to be comparable to levels of IgM found in mouse plasma (0.22 ±0.036 mg/L). IgM binding was detected by a 30-minute incubation with the following IgM μ chain specific antibodies: FITC mouse anti-human IgM (1:5, Clone: G20-127, BD Pharmingen) and APC rat anti-mouse IgM (1:100, Clone ID: II/41, BD Pharmingen) diluted in 1% BSA. Annexin-V and PI staining was then performed.

### Annexin-V (AnnV) and Propidium Iodide (PI) staining

PtdSer exposure was detected using Alexa Fluor-647, FITC or PE conjugated Annexin-V (all 1:100, BioLegend, UK) diluted in AnnV binding buffer (Hanks Balanced Salt Solution H6648 with 2.5mL 1M CaCl_2_) and incubated for 15-minutes. PI was used as above. Cells were designated non-apoptotic (AnnV^-^PI^-^), early (AnnV^+^PI^-^) or late (AnnV^+^PI^+^) apoptotic. Staining was assessed by flow cytometry on a BD FACSCalibur (10,000 events collected) and analysed using FlowJo (TreeStar).

### FACS

Thymocytes, treated and stained as above, were sorted on a BD FACSAria II with a 70μM nozzle. Post-sorting checks were performed to ensure populations remained in original FSC and SSC regions and to assess staining.

### Confocal microscopy

Sorted populations of thymocytes were treated with plasma and stained as above, with the following modifications. Prior to apoptosis induction thymocytes were stained with CellTracker Green CMFDA (Invitrogen) according to manufactures instructions. Texas Red goat anti-mouse μ chain specific IgM (SouthernBiotech) was used and samples were stained with DAPI (Invitrogen) and PI staining was omitted. Jurkat cells were treated and stained as above with the addition of DAPI and omission of PI staining. Samples were mounted using ProLong Gold antifade reagent (Life Technologies) and viewed using a Leica SP5 with an x63/1.4 objective. Single stains were used to check for spectral-bleed through. Whole image contrast/brightness and pseudocolour was adjusted using ImageJ (NIH) or Adobe Photoshop CS5.

### Transmission Electron Microscopy (TEM)

Sorted thymocytes were fixed in 3% glutaraldehyde in 0.1M sodium cacodylate buffer, pH 7.3, for 2-hours and washed thrice in 0.1M sodium cacodylate for 10-minutes each. Post-fixation was performed by a 45-minute incubation in 1% osmium tetroxide in 0.1M sodium cacodylate before samples were washed thrice in 0.1M sodium cacodylate for 10-minutes each. Samples were dehydrated by 10-minute incubations in 50%, 70%, 90% and 100% normal grade acetones before a further two 10-minute washes in analar acetone. Samples were embedded in Araldite resin and 1μM sections cut on a Reichert OMU4 ultramicrotome. Sections were stained with Toluidine Blue and suitable areas for investigation selected by light microscopy. From selected areas 60nM ultrathin sections were cut and stained with uranyl acetate and lead citrate before viewing in a Philips CM120 transmission electron microscope. Images were taken on a Gatan Orius CCD camera. Whole image contrast/brightness was adjusted using ImageJ.

### Mouse plasma generation and erythrocyte isolation

Total blood was collected via cardiac puncture from terminally anesthetized mice into Multivette 600 Lithium Heparin coated tubes (Sarstedt). Plasma was collected following centrifugation at 1500 x *g* for 10-minutes. The buffy coat was discarded and erythrocyte pellet was washed in PBS and centrifuged at 600 *x g* for 10-minutes thrice before being diluted x3.5 in PBS.

### Permeabilisation and assessment of IgM binding to mouse erythrocytes

Isolated erythrocytes (200μL) were stained with Alexa Fluor-488 rat anti-mouse Ter-119/Erythroid cells antibody (1:100, BioLegend) for 30-minutes before being washed with PBS and centrifuged at 600 *x g* for 10-minutes. Permeabilisation was achieved by a 10-minute incubation in 0.05% saponin after which erythrocytes were washed. Erythrocytes were incubated in PBS or Balb/c or RAG1-deficient mice plasma at a ratio of 1:1 (erythrocyte volume:plasma volume) for 30-minutes before being washed. APC Mouse (Balb/c) IgM κ isotype control was used as control irrelevant mouse IgM antibodies. IgM binding was detected by a 30-minute incubation with APC rat anti-mouse IgM. AnnV staining was performed as above to confirm permeabilisation. Samples were diluted in AnnV binding buffer before staining was assessed by flow cytometry on a BD FACSCalibur. Erythrocytes were identified by Ter-119 positivity (defined by isotype controls) and 5000–10000 Ter-119^+^ events were collected and analysed using FlowJo.

### Statistical analysis

Results expressed as means ±SEM. Statistical tests used: student *t*-test, Kruskal-Wallis one-way ANOVA or two-way ANOVA with Dunn’s or Bonferroni multiple comparisons test when appropriate. *P* < 0.05 was considered to be statistically significant. Statistical analysis performed using Prism 6 (GraphPad Software, San Diego).

## Results

### Circulating mouse IgM preferentially bind a distinct subpopulation of AnnV^+^PI^+^ thymocytes

**Fig 1:**
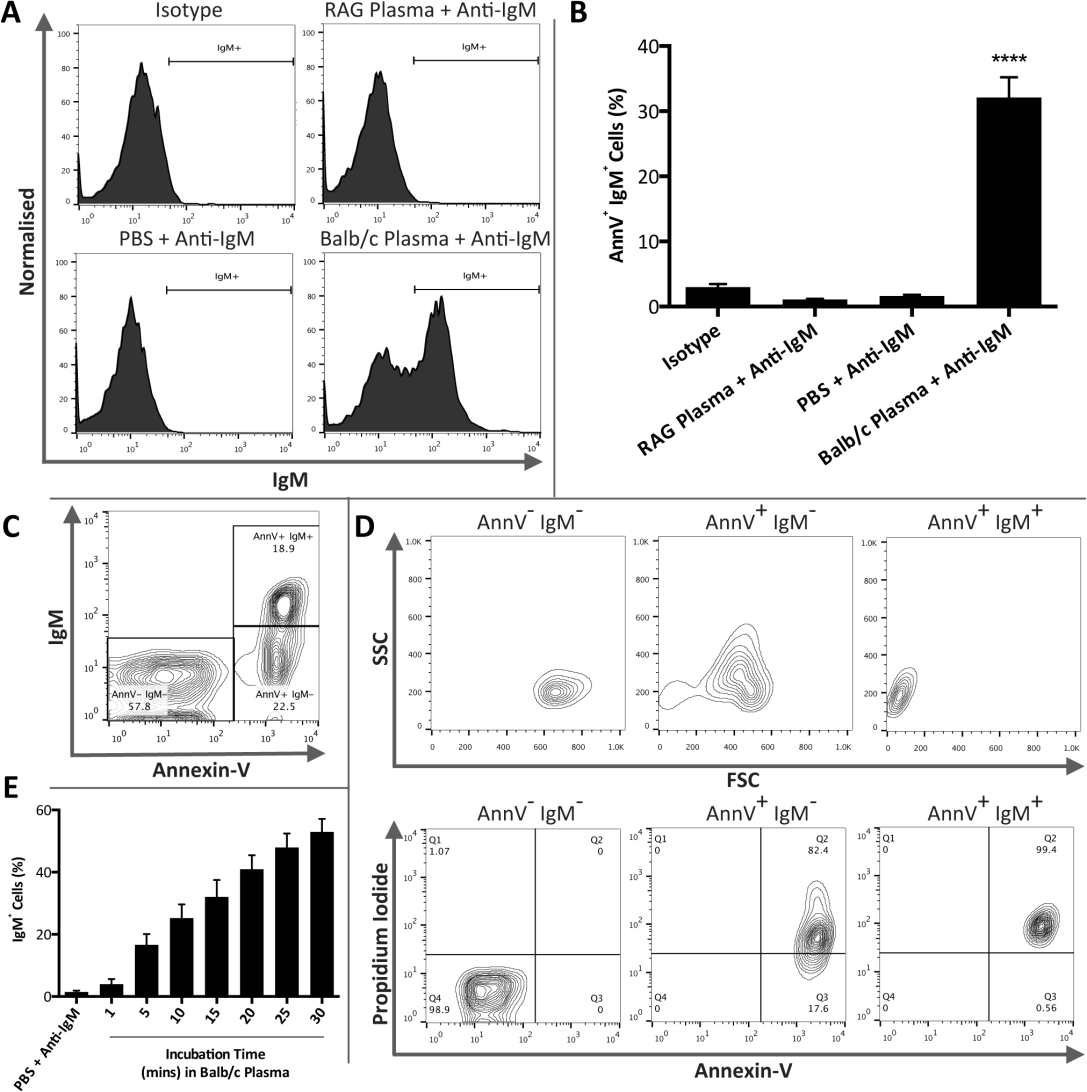
IgM rapidly and specifically binds to a distinct population of AnnV^+^PI^+^ mouse thymocytes. Mouse thymocytes were rendered apoptotic by overnight culture. Thymocytes were then exposed to either mouse anti-human isotype control antibodies (mouse (Balb/c) IgM κ isotype control), PBS, Balb/c plasma (as a source of IgM) or plasma from immunodeficient RAG1-deficient mice. IgM binding was then assessed by flow cytometry. Annexin-V (AnnV) and propidium iodide (PI) staining, assessed by flow cytometry, was used to determine the level of non-apoptotic (AnnV^**-**^PI^**-**^), early (AnnV^**+**^PI^**-**^) or late apoptotic (AnnV^**+**^PI^**+**^) thymocytes. **A**) Representative histograms illustrating IgM binding to AnnV^**+**^ apoptotic thymocytes within treatment groups. **B**) Apoptotic thymocytes were gated on the basis of AnnV positivity and the percentage of AnnV^**+**^IgM^**+**^ thymocytes was assessed. IgM binding in isotype, RAG1-deficient mice plasma and PBS treatment groups remained non-significant whilst samples exposed to Balb/c plasma exhibited significant binding (Kruskal–Wallis one-way ANOVA; *P* < 0.0001). However, surprisingly approximately 68% were IgM^**-**^. **C**) Samples exposed to Balb/c plasma consisted of three distinct populations: AnnV^**-**^IgM^**-**^, AnnV^**+**^IgM^**-**^ and AnnV^**+**^IgM^**+**^. **D**) The three distinct populations present in samples exposed to Balb/c plasma were further characterised by examining the FSC/SSC profile and AnnV and PI positivity. Although both AnnV^**+**^IgM^**-**^ and AnnV^**+**^IgM^**+**^ populations were predominantly AnnV^**+**^ PI^**+**^ they occupied distinct separate regions of FSC/SSC contour plots. **E**) Within the AnnV^**+**^IgM^**+**^ population IgM binding occurred rapidly (*n* = 4). Data representative of *n* = 5 isotype, *n* = 6 RAG1 deficient mice plasma + Anti-IgM, *n* = 8 PBS + Anti-IgM and *n* = 8 Balb/c plasma + Anti-IgM. Data expressed as means ±SEM.

We examined IgM binding to mouse thymocytes rendered apoptotic by ageing overnight as this method elicited mixed levels of apoptosis thereby allowing the assessment of IgM binding to thymocytes at variable stages of apoptosis. Thymocytes were exposed to either PBS, Balb/c plasma (as a source of circulating IgM) or plasma from RAG1-deficient mice, which are deficient in both IgG and IgM [64–66]. Previous work indicates that IgM binds late apoptotic cells [26, 36, 47–49], we therefore focused our attention on AnnV^+^ thymocytes and assessed the percentage of AnnV^+^IgM^+^ thymocytes (Fig 1A and 1B). IgM binding of thymocytes by control isotype antibody or following incubation in RAG1-deficient mice plasma or PBS remained non-significant whilst samples exposed to Balb/c plasma exhibited significant binding with 32.1 ±3.1% of cells being AnnV^+^IgM^+^ (Kruskal–Wallis one-way ANOVA; *P* < 0.0001; *n* = 5 isotype, *n* = 6 RAG1-deficient mice plasma + Anti-IgM, *n* = 8 PBS + Anti-IgM and Balb/c plasma + Anti-IgM) (Fig 1B). Additional experiments were performed using an unconjugated IgM isotype control that also exhibited no binding to AnnV^+^ mouse thymocytes (S1 Fig). Approximately 68% of AnnV^+^ cells were IgM^-^ and further examination of the cell populations present within samples exposed to Balb/c plasma revealed three distinct populations: AnnV^-^IgM^-^, AnnV^+^IgM^-^ and AnnV^+^IgM^+^ cells (Fig 1C). Analysis of the FSC/SSC contour plots of these populations revealed contrasting profiles, yet comparable AnnV/PI staining (Fig 1D). As expected the AnnV^-^IgM^-^ population exhibited no PI positivity. Interestingly, AnnV^+^IgM^-^ and AnnV^+^IgM^+^ populations demonstrated disparate FSC/SSC profiles, yet both populations were predominantly AnnV^+^PI^+^. Apoptotic thymocytes incrementally incubated in Balb/c plasma indicated that circulating IgM bound rapidly to cells localized within the region defined as AnnV^+^IgM^+^ on FSC/SSC contour plots (Fig 1E). The absence of IgG binding to AnnV^+^ thymocytes confirmed that this preferential binding was not due to non-specific Ig interaction (S2 Fig).

### Circulating IgM bind to mouse apoptotic thymocytes with evidence of a disrupted cell membrane

**Fig 2:**
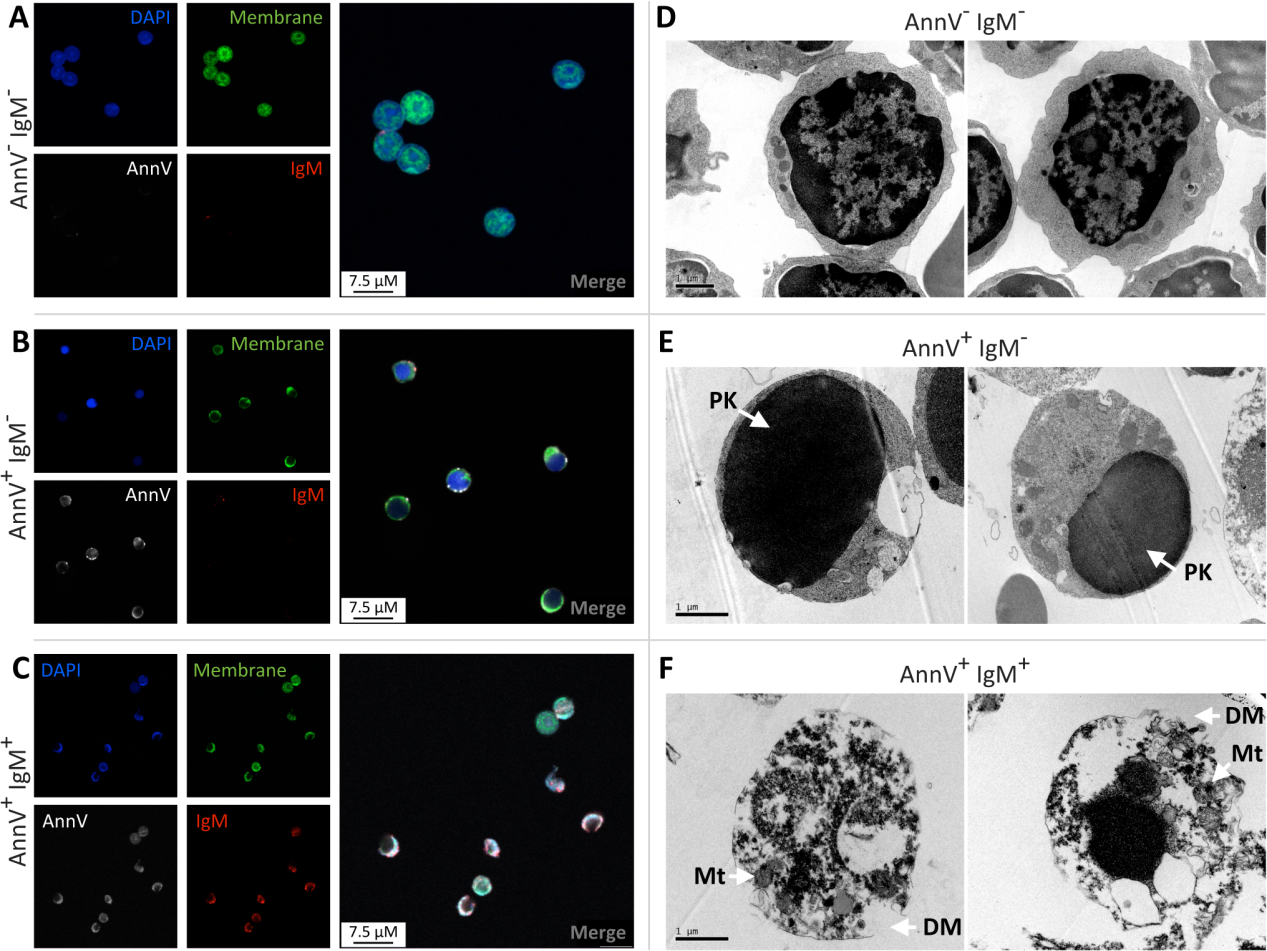
IgM bound mouse apoptotic thymocytes appear disrupted. Mouse thymocytes were stained with a membrane cell tracker and then rendered apoptotic by overnight culture. Thymocytes were then exposed to Balb/c plasma prior to incubation with anti-IgM, Annexin-V (AnnV) and DAPI. On the basis of FSC/SSC thymocytes were then sorted into AnnV^**-**^IgM^**-**^ (**A, D**), AnnV^**+**^IgM^**-**^ (**B, E**) and AnnV^**+**^IgM^**+**^ (**C, F**) populations, as illustrated in Fig 1D, by FACS and examined by confocal or electron microscopy. **A**) AnnV^**-**^IgM^**-**^ thymocytes were AnnV and IgM negative. **B**) AnnV^**+**^IgM^**-**^ thymocytes exhibited AnnV membrane staining but remained IgM negative and morphologically appeared intact. **C**) AnnV^**+**^IgM^**+**^ thymocytes were AnnV positive and appeared disrupted with an apparent IgM^**+**^ staining. **D**) AnnV^**-**^IgM^**-**^ cells appeared non-apoptotic with normal nuclei and intact plasma membranes. **E**) AnnV^**+**^IgM^**-**^ cells exhibited nuclear pyknosis (PK, arrowed) with preservation of cytoplasmic and membrane integrity. **F**) Electron microscopy studies confirmed that AnnV^**+**^IgM^**+**^ cells had a disrupted membrane (DM, arrowed) with cellular disorganization, but retention of mitochondria (Mt, arrowed). Representative images taken using either a Leica SP5 with an x63/1.4 objective or a Philips CM120 transmission electron microscope with a Gatan Orius CCD camera. Whole image contrast/brightness was adjusted using ImageJ or Adobe Photoshop.

We explored whether apoptotic thymocytes that bound IgM demonstrated any morphological features that might explain the disparity of IgM binding to AnnV^+^PI^+^ cells and the differing FSC/SSC profiles of the AnnV^+^IgM^+^ and AnnV^+^IgM^-^ cell populations. CellTracker Green labelled thymocytes were sorted into AnnV^-^IgM^-^, AnnV^+^IgM^-^ and AnnV^+^IgM^+^ populations by FACS and examined by confocal microscopy. Unsurprisingly AnnV and IgM staining was absent in sorted AnnV^-^IgM^-^ cell populations (Fig 2A). Similarly, AnnV^+^IgM^-^ cells only displayed AnnV staining that appeared restricted to the membrane with no obvious morphological differences to AnnV^-^IgM^-^ cells (Fig 2B). Some AnnV^+^IgM^+^ cells appeared disrupted (Fig 2C) and therefore electron microscopy was used to investigate the cellular and membrane morphology in detail (Fig 2D–2F). AnnV^-^IgM^-^ cells (Fig 2D) appeared non-apoptotic with an intact membrane whilst AnnV^+^IgM^-^ cells (Fig 2E) exhibited nuclear pyknosis with preservation of cytoplasmic and membrane integrity. However, the membrane of AnnV^+^IgM^+^ cells (Fig 2F) was severely disrupted compared to either AnnV^-^IgM^-^ cells and AnnV^+^IgM^-^ cells. Despite marked membrane disruption AnnV^+^IgM^+^ cells appeared to maintain sufficient cellular integrity to prevent the overt loss of organelles, such as mitochondria as evident in Fig 2F. Additional electron microscopic images are provided as supporting information (S3 Fig).

### Circulating IgM binds strongly to saponin permeabilised mouse thymocytes

**Fig 3:**
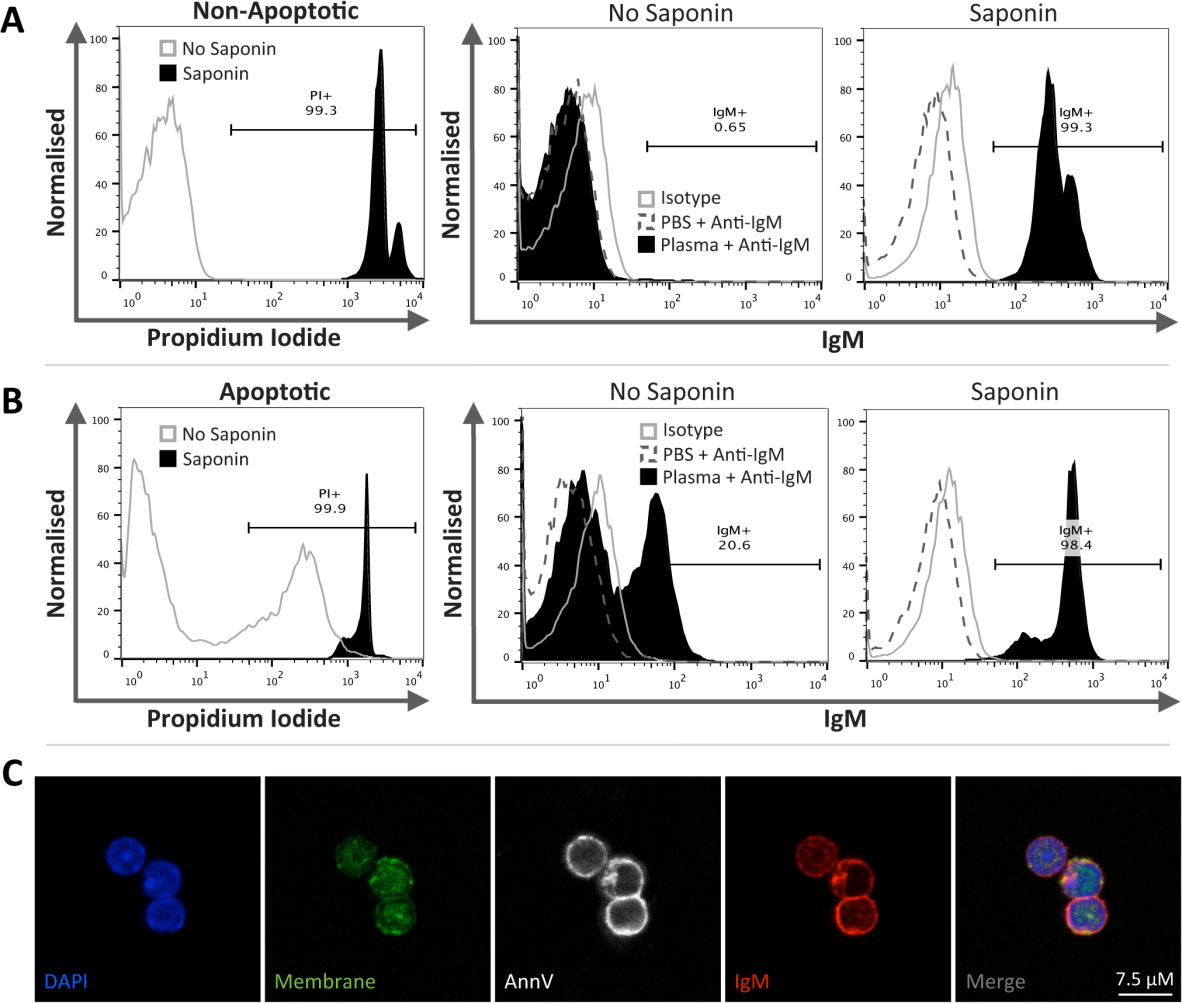
IgM binds strongly to permeabilised non-apoptotic and apoptotic thymocytes. Non-apoptotic and apoptotic mouse thymocytes, induced by overnight culture, were permeabilised with saponin prior to incubation with Balb/c plasma. Permeability was confirmed by PI staining and IgM binding was assessed by flow cytometry. For confocal microscopy AnnV staining was used to indicate permeabilisation **A**) Following permeablisation non-apoptotic thymocytes were predominantly PI positive. Non-permeable non-apoptotic thymocytes remained consistently IgM negative, whilst the majority of permeable thymocytes were IgM positive. Inclusion of isotype control IgM antibodies (mouse (Balb/c) IgM κ isotype control) confirmed that IgM binding was not due to a non-specific interaction of IgM. **B**) A small proportion of apoptotic thymocytes were PI positive representing late apoptotic thymocytes; however following permeabilisation with saponin the cell population became PI positive. Whilst a minority of apoptotic thymocytes were IgM positive, representing the AnnV^**+**^IgM^**+**^ population discussed in Fig 1C–1E, with permeabilisation the population became IgM positive. **C**) Confocal microscopy demonstrates that permeabilised thymocytes were AnnV positive, used to indicate permeability, with IgM appearing to bind part of the cellular membrane. Representative histograms (Non-apoptotic thymocytes *n* = 3; Apoptotic thymocytes *n* = 4). Images were taken using a Leica SP5 with an x63/1.4 objective. Whole image contrast/brightness was adjusted using ImageJ or Adobe Photoshop.

In light of the previous results we hypothesized that circulating IgM may be interacting with intracellular epitopes only accessible following significant plasma membrane integrity loss and that these epitopes were not generated during apoptosis by caspases and other enzymes. We examined whether IgM bound non-apoptotic thymocytes, which are consistently IgM negative, following permeabilisation with saponin. IgM binding of permeabilised non-apoptotic thymocytes was assessed following exposure to PBS, or plasma from either Balb/c or RAG1-deficient mice (*n* = 3 per group). For flow cytometry analysis PI staining was used to indicate permeabilisation. Saponin treated non-apoptotic thymocytes were PI^+^ indicating successful permeabilisation and demonstrated a dramatic shift in IgM positivity whilst non-saponin treated non-apoptotic thymocytes remained negative for both PI and IgM (Fig 3A). We next assessed if IgM would bind more strongly to saponin treated apoptotic thymocytes, a large percentage of which remain IgM negative as evident in Fig 1 (*n* = 4 per group). A small proportion of apoptotic thymocytes were PI positive representing late apoptotic thymocytes but nearly all cells became PI positive (>99%) following permeabilisation with saponin (Fig 3B). In the absence of permeabilisation a minority of apoptotic thymocytes were IgM^+^ representing the AnnV^+^IgM^+^ population shown In Fig 1C–1E. However, following permeabilisation the majority of cells (>98%) became IgM positive (Fig 3B). Inclusion of isotype control IgM antibodies confirmed that IgM binding of permeabilised cells was not due to a non-specific interaction (Fig 3A and 3B). Furthermore, confocal microscopy confirmed these results as permeabilised non-apoptotic thymocytes were AnnV^+^, indicating permeability, and IgM^+^ with IgM appearing to bind in the region of the cellular membrane (Fig 3C). Note that for confocal microscopy AnnV staining was used to indicate permeabilisation.

### Circulating IgM in human serum binds AnnV^+^PI^+^ and saponin permeabilised human Jurkat cells

**Fig 4:**
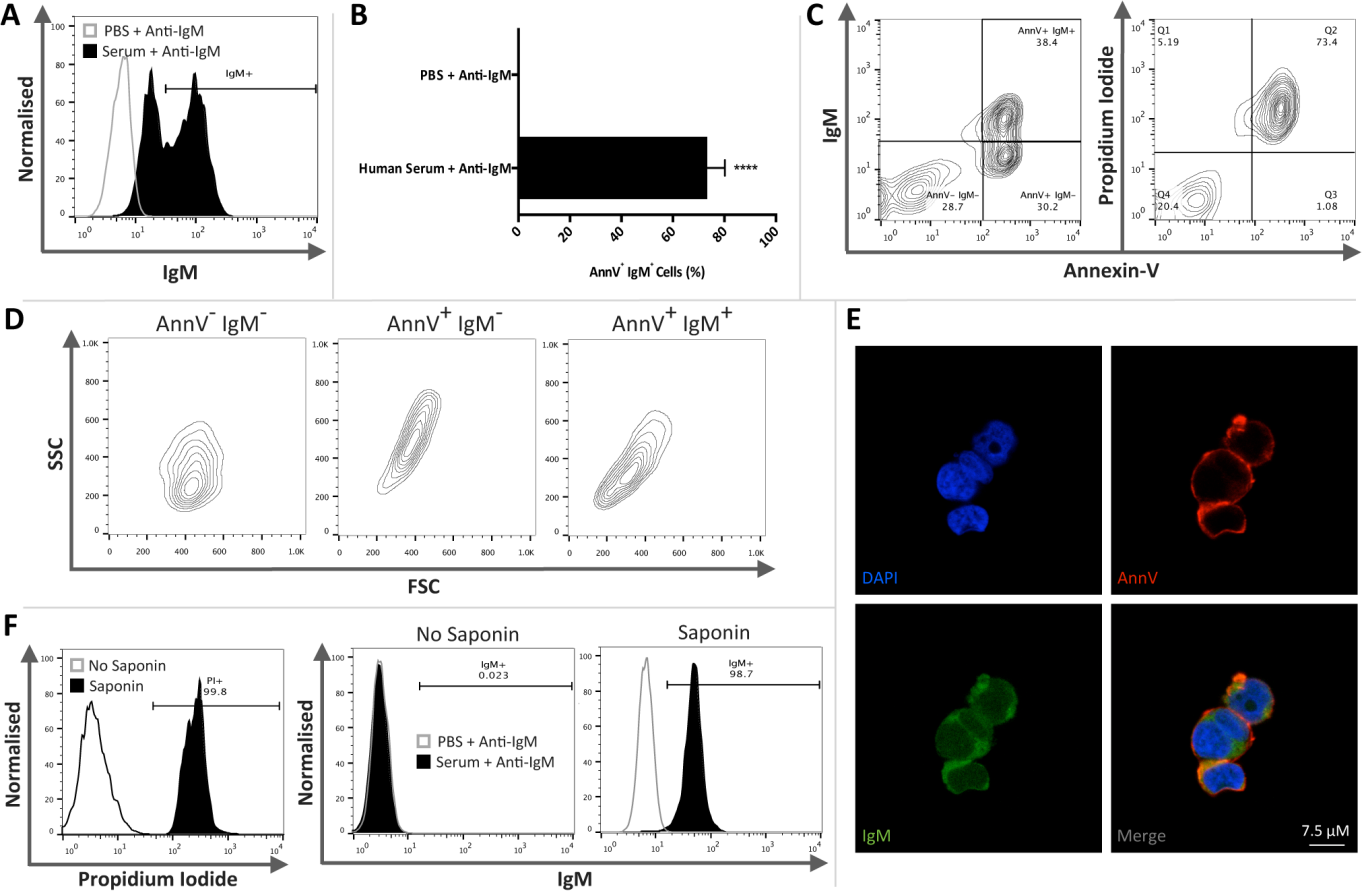
IgM binds AnnV^+^ PI^+^ and permeabilised human Jurkat cells. Human Jurkat cells were treated with camptothecin for 16-hours to induce apoptosis. Jurkat cells were then exposed to PBS or human serum as a source of IgM. IgM binding was then assessed by flow cytometry. Annexin-V (AnnV) and PI staining, assessed by flow cytometry, was used to determine the level of non-apoptotic (AnnV^**-**^PI^**-**^), early (AnnV^**+**^PI^**-**^) or late apoptotic (AnnV^**+**^PI^**+**^) thymocytes. **A**) Representative histogram illustrating IgM binding to AnnV^**+**^ apoptotic Jurkat cells within human serum and PBS treatment groups. **B**) Apoptotic Jurkat cells were gated on the basis of AnnV positivity and the percentage of AnnV^**+**^IgM^**+**^ Jurkat cells was assessed. Apoptotic Jurkat cells exposed to human serum exhibited significant IgM binding. (Student’s *t*-test; *P* < 0.0001; *n* = 4) **C**) Samples exposed to human plasma consisted of three distinct populations: AnnV^**-**^IgM^**-**^, AnnV^**+**^IgM^**-**^ and AnnV^**+**^IgM^**+**^. Apoptotic Jurkat cells were largely AnnV^**+**^PI^**+**^. **D**) The three distinct populations present in samples exposed to human serum were further characterised by examining FSC/SSC. Although both AnnV^**+**^IgM^**-**^ and AnnV^**+**^IgM^**+**^ populations were AnnV^**+**^PI^**+**^ they occupy distinct separate regions of FSC/SSC contour plots. **E**) Confocal microscopy revealed that late apoptotic Jurkat cells exhibited AnnV and IgM staining which appeared to be restricted to the membrane. **F**) Non-apoptotic Jurkat cells were permeabilised with saponin prior to incubation with human serum. Permeability was confirmed by PI staining and IgM binding was assessed by flow cytometry. Following permeabilisation non-apoptotic Jurkat cells were predominantly PI positive. Non-permeable non-apoptotic Jurkat cells remained consistently IgM negative, whilst permeable Jurkat cells were IgM positive. Representative histograms and contour plots (*n* = 3 per group). Images were taken using a Leica SP5 with an x63/1.4 objective. Whole image contrast/brightness was adjusted using ImageJ or Adobe Photoshop. Data expressed as means ±SEM.

The relevance of these findings to human biology was explored using human Jurkat cells to determine if they demonstrated a similar IgM binding profile to that observed in mouse thymocytes. Jurkat cells were treated with camptothecin for 16-hours to induce apoptosis and exposed to PBS or human serum as a source of IgM. Apoptotic Jurkat cells were gated on the basis of AnnV positivity and the percentage of AnnV^+^IgM^+^ Jurkat cells was determined (Fig 4A–4C). A significant proportion of apoptotic Jurkat cells exposed to human serum were IgM^+^ (Student’s *t*-test; *P* < 0.0001; *n* = 4) compared to those incubated in PBS (Fig 4A). As observed in murine apoptotic thymocytes, Jurkat cells exposed to human serum also consisted of three distinct populations (Fig 4C) including an AnnV^-^IgM^-^ population that comprised of non-apoptotic cells. In addition, despite all AnnV^+^ cells being PI^+^, two AnnV^+^ populations were evident being either AnnV^+^IgM^-^ or AnnV^+^IgM^+^. These AnnV^+^IgM^-^ and AnnV^+^IgM^+^ cell populations occupied relatively distinct regions of FSC/SSC contour plots (Fig 4D), although the downward shift of AnnV^+^IgM^+^ cells in relation to AnnV^+^IgM^-^ cells was subtler than that observed in murine thymocytes (Fig 1D). Confocal microscopy revealed that apoptotic Jurkat cells exhibited AnnV and IgM positive staining (Fig 4E). IgM binding to saponin permeabilised non-apoptotic Jurkat cells was assessed to determine if human IgM could bind intracellular epitopes within normal cells (*n* = 3 per group). For flow cytometry analysis PI staining was used to indicate permeabilisation. Following permeablisation non-apoptotic Jurkat cells were predominantly PI and IgM positive (>98%), whilst non-saponin treated non-apoptotic Jurkat cells remained consistently PI and IgM negative (Fig 4F). Apoptotic Jurkat cells exposed to Balb/c plasma also exhibited positive IgM binding indicating that mouse IgM was able to bind human cells thereby supporting the broad nature of antigens recognised and bound by IgM (S4 Fig).

### Circulating IgM in human serum binds saponin permeabilised human proximal tubular epithelial cells

**Fig 5:**
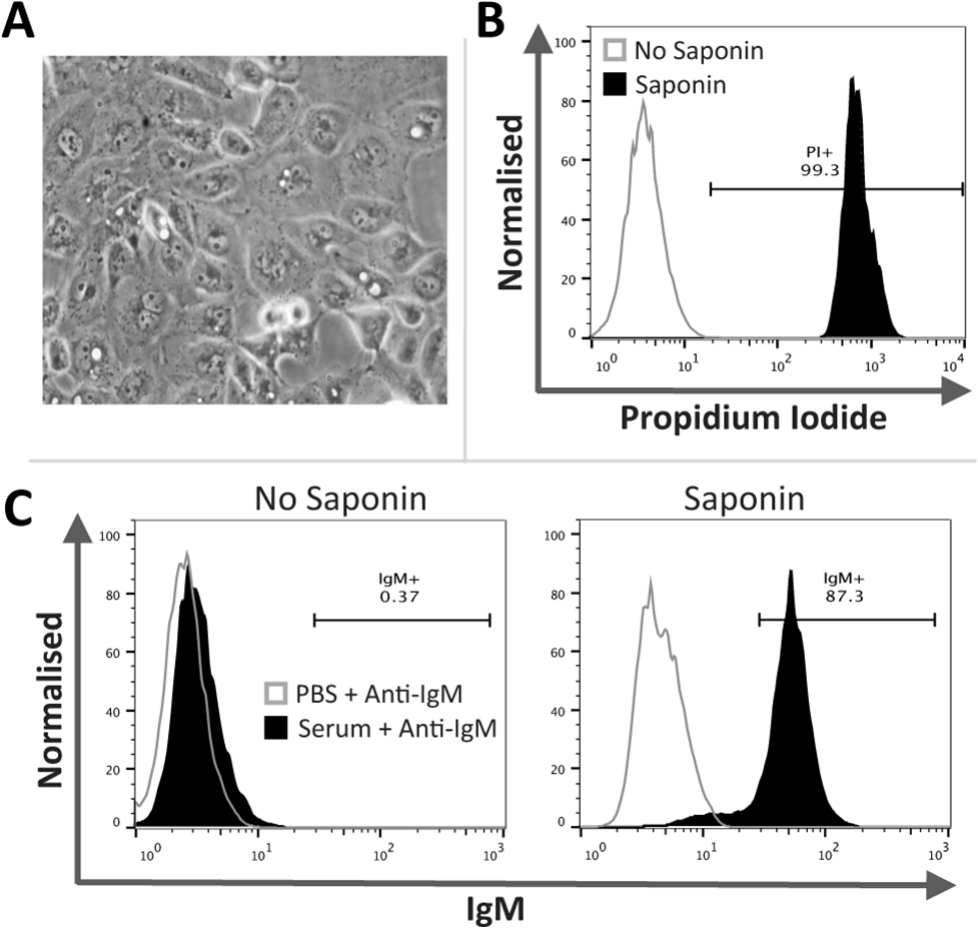
IgM binds to permeable human proximal tubule epithelial (HK-2) cells. Non-apoptotic HK-2 cells (**A**) were permeabilised with saponin prior to incubation with human serum (as a source of IgM). Permeability was confirmed by PI staining and IgM binding was assessed by flow cytometry. **B**) Saponin treated HK-2 cells demonstrated a distinct shift in PI positivity indicating successful permeabilisation. **C**) Non-permeable HK-2 cells remained consistently IgM negative, whilst HK-2 cells treated with saponin were IgM positive. Representative histograms and contour plots (*n* = 3 per group). Images were taken using a Zeiss Axiovert S100 with an x32/0.4 objective and CoolSnap RS Photometrics camera. Whole image contrast/brightness was adjusted using ImageJ.

To determine if circulating IgM could interact with intracellular antigens present within non-lymphoid cells we examined IgM binding to saponin permeabilised human proximal tubular epithelial (HK-2) cells; a cell line derived from normal adult human kidney (Fig 5A). A distinct shift in PI positivity confirmed permeablisation of saponin treated HK-2 cells (Fig 5B). Control non-permeabilised HK-2 cells remained consistently IgM^-^ whilst permeabilised HK-2 cells were IgM^+^ (Fig 5C) (*n* = 3 per group).

### Circulating mouse IgM specifically binds permeabilised erythrocytes

**Fig 6:**
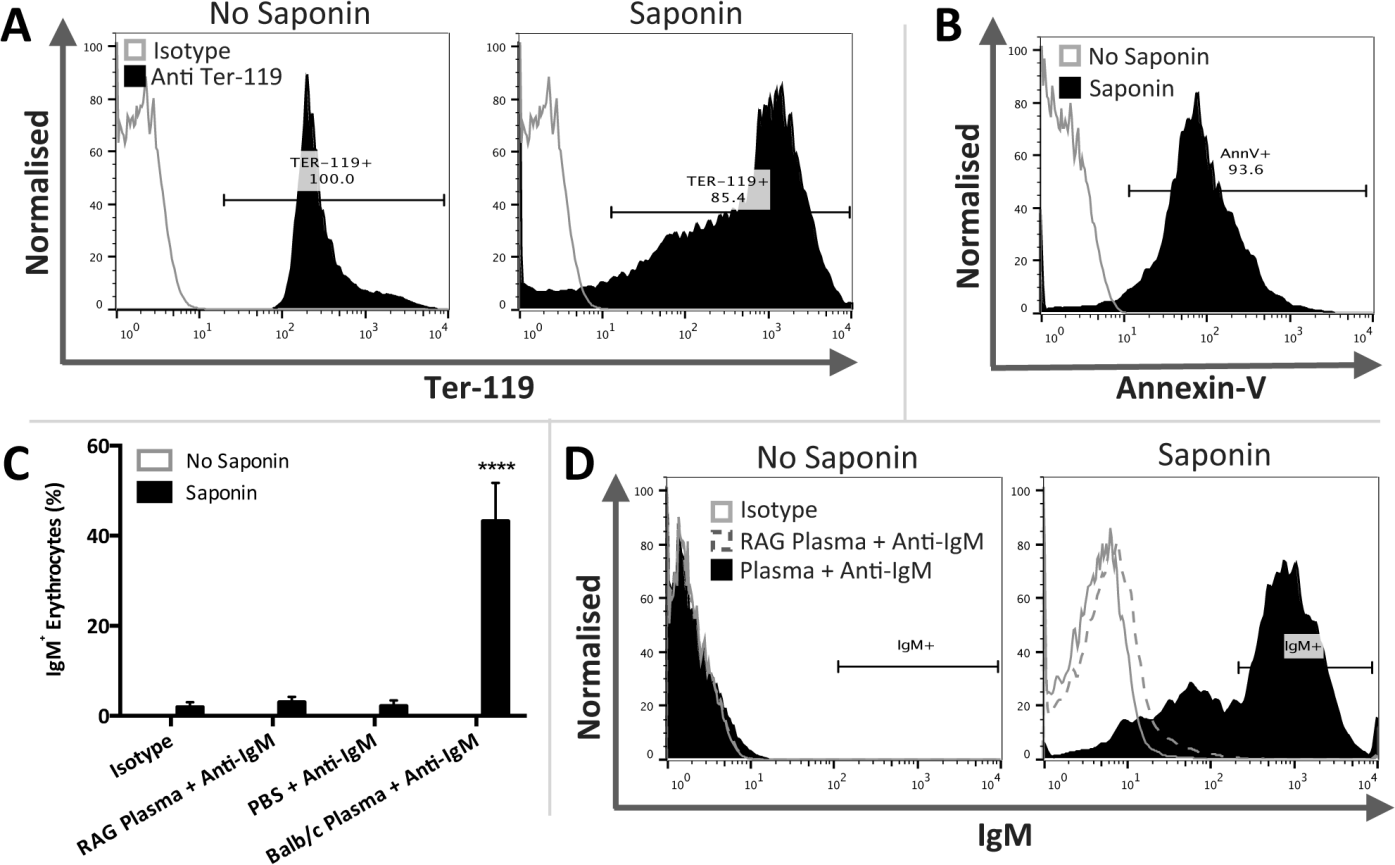
IgM binds permeabilised mouse erythrocytes. Mouse erythrocytes were stained with erythrocyte marker Ter-119 prior to permeabilisation with saponin. Annexin-V (AnnV) staining was used to confirm permeability. Non-permeable and permeable erythrocytes were then exposed to either mouse IgM isotype control antibodies, PBS, Balb/c plasma (as a source of IgM) or plasma from immunodeficient RAG1-deficient mice. Staining was assessed by flow cytometry. **A**) Both non-permeable and permeable erythrocytes were identified and gated on the basis of Ter-119 positivity. **B**) Permeable Ter-119^**+**^ erythrocytes demonstrated a distinct shift in AnnV positivity indicating successful permeabilisation. **C**) Non-permeable and permeable erythrocytes were gated on the basis of Ter-119 positivity and the percentage of IgM^**+**^ erythrocytes was assessed. IgM binding to non-permeable and permeable erythrocytes in isotype, RAG1-deficient mice plasma and PBS treatment groups remained non-significant. Permeable erythrocytes exposed to Balb/c plasma exhibited significant binding (two-way ANOVA; *P* < 0.0001; *n* = 4 per group). **D**) Representative histograms illustrating IgM binding to non-permeable and permeable Ter-119^**+**^ erythrocytes within the treatment groups. Data expressed as means ±SEM.

Although confocal microscopy studies of AnnV^+^IgM^+^ apoptotic thymocytes and apoptotic Jurkat cells suggested IgM binding to the cell membrane it was possible that IgM bound intracellular organelles. We therefore used mouse erythrocytes as an anucleate target with no intracellular organelles and determined if circulating IgM bound permeabilised erythrocytes. Mouse erythrocytes were studied due to the availability of both control mouse IgM isotype antibodies and RAG1-deficient mice plasma to evaluate the specificity of IgM binding. Erythrocytes were stained with the erythrocyte marker Ter-119 prior to permeabilisation with saponin. Ter-119 positivity was used to identify erythrocytes (Fig 6A). Successful permeabilisation of saponin treated erythrocytes was confirmed by AnnV positivity (Fig 6B) and the percentage of Ter-119^+^ IgM^+^ erythrocytes was assessed (Fig 6C and 6D). IgM binding to control and saponin treated erythrocytes in isotype, RAG1-deficient mice plasma and PBS treatment groups remained non-significant whilst permeabilised erythrocytes exposed to Balb/c plasma exhibited significant binding with 43.1 ±8.4% IgM^+^ erythrocytes (two-way ANOVA; *P* < 0.0001; *n* = 4).

## Discussion

Circulating IgM antibodies play a non-redundant role in apoptotic cell clearance, evident by the development of autoimmune disease in IgM-deficient mice [31, 32]. IgM binding to late apoptotic cells [26, 36, 47–49] leads to complement activation and subsequent opsonisation of IgM^+^ cells by complement components that may be recognised by phagocytes [25, 28–30]. In this paper we carefully explored the binding of circulating IgM to apoptotic cells, both human and mouse, at different apoptotic stages. We introduce the concept that IgM antibody binding requires significant plasma membrane disruption thereby allowing access to cytoplasmic or cell membrane antigens.

**Fig 7:**
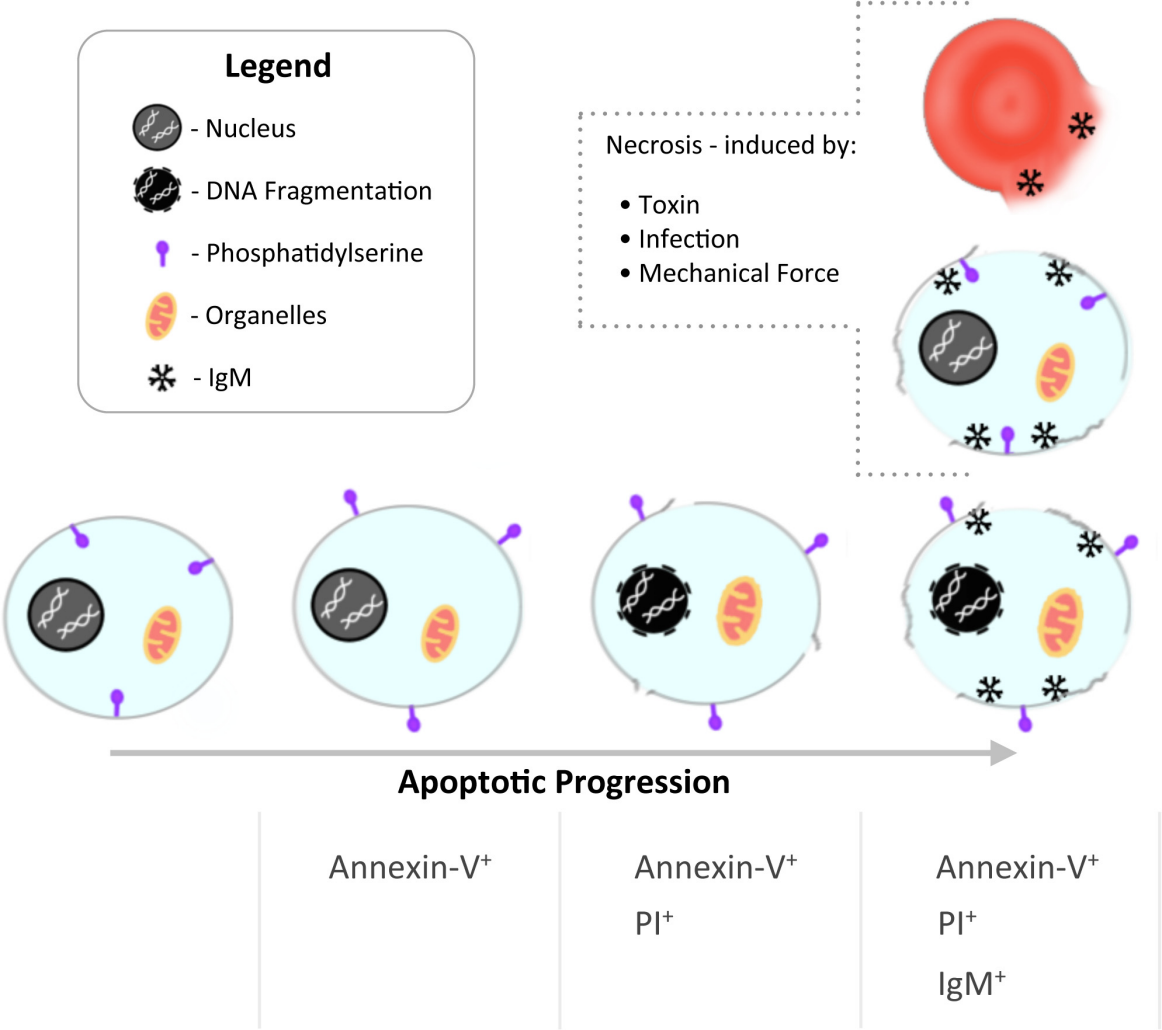
Schematic diagram of IgM binding to apoptotic or disrupted cells. As cells progress through apoptosis IgM binding occurs following phosphatidylserine exposure (Annexin-V binding) and loss of membrane integrity (PI binding). At this point IgM is able to bind intracellular cell membrane or cytoplasmic antigens. Similarly, IgM may bind any cells including lymphocytes or erythrocytes that have lost cellular integrity and have become permeable following cell necrosis or fragmentation.

IgM antibodies preferentially bind late apoptotic cells [26, 36, 47–49]. A key finding of this work is that IgM binds a subpopulation of AnnV^+^PI^+^ apoptotic thymocytes with IgM^+^ cells exhibiting a different FSC/SSC profile to AnnV^+^PI^+^ apoptotic thymocytes that were IgM^-^. These data suggested a fundamental difference in the properties of IgM^+^AnnV^+^PI^+^ apoptotic thymocytes, with potential epitopes bound by IgM either being absent or inaccessible in IgM^-^AnnV^+^PI^+^ thymocytes. Apoptosis consists of multiple temporal stages defined by morphological changes such as nuclear pyknosis, vacuolation, cell shrinkage and the presence or absence of plasma membrane integrity [4–6]. Confocal and electron microscopy imaging analysis of FACS sorted cells suggested that IgM may bind the cell membrane of AnnV^+^IgM^+^, with the membrane of said cells exhibiting marked disruption with obvious ‘holes’ evident. This contrasted IgM negative AnnV^+^PI^+^ cells, which retained a predominantly intact cell membrane. These profound morphological changes are likely to account for their different FSC/SSC profile evident on flow cytometry. The relevance of our carefully controlled mouse IgM binding data to human cells was provided by similar findings in studies of apoptotic Jurkat cells. These flow cytometry and morphological studies of IgM^+^ or IgM^-^ apoptotic cells suggest that the binding of IgM (MW 970kDa) requires physical access to the cellular interior via a severely disrupted cell membrane and that such access is not provided by the loss of cell membrane integrity that is defined by positive staining with propidium iodide (MW 0.67kDa). This model is schematically depicted in Fig 7 in which apoptotic cells undergo a progressive and increasing loss of cell membrane integrity that culminates in large pentameric IgM [67, 68] molecules being able to access and bind internal epitopes.

A key question concerned the nature of the epitopes recognized by IgM and whether these epitopes were generated *de novo* during apoptosis by caspases or other enzymes, or by other forms of cell stress, such as oxidation. Following saponin permeabilisation both human and mouse thymocytes became strongly IgM^+^, irrespective of whether they were non-apoptotic or apoptotic. Also, human renal tubular (HK-2) cells became strongly IgM^+^ following permeabilisation indicating that the presence of intracellular epitopes capable of IgM recognition is not confined to lymphoid cells. Furthermore, we found that IgM bound a significant proportion of permeabilised mouse erythrocytes. Erythrocytes are anucleate with few organelles and a biochemically less complicated cell membrane compared to nucleated cells, such as lymphocytes. These data suggested that at least a proportion of epitopes recognized and bound by IgM within this study are cell membrane associated. IgM antibodies bind a diverse range of epitopes, although it is clear that studies predominantly report binding to membrane phospholipids that are modified during apoptosis such as by oxidation [27, 36, 41, 50–52, 54–59]. Our data suggests that IgM bound epitopes, such as membrane phospholipids, do not need to have been modified during apoptosis for IgM to bind. This is quite a pertinent finding as work by Kim *et al*., (2002) indicates that IgM binding occurs following the activation of a specific phospholipase (iPLA2). This phospholipase remodels apoptotic cell membranes exposing IgM bound phospholipids. This further supports the concept that IgM binding is restricted by accessibility to IgM bound epitopes.

These data indicate that IgM is able to recognize and bind epitopes present within both normal and apoptotic nucleated cells thereby suggesting that IgM bound epitopes are not generated by the activation of caspase cascades associated with apoptosis. Indeed, the presence of such apoptosis-independent epitopes within normal cells could provide the substrate for IgM binding to cells that have undergone any form of cell lysis such as primary necrosis secondary to ischaemia etc. or cell injury induced by granzyme/perforin or complement (Fig 7). The binding of IgM to permeabilised erythrocytes is intriguing as previous work has shown that natural autologous IgG1 antibodies and complement are implicated in the recognition and clearance of senescent erythrocytes [69]. Furthermore, natural IgM antibodies have been shown to bind exosomes derived from reticulocytes [70]. There is little data available regarding the clearance of the fragmented normal erythrocytes generated in patients with conditions such as microangiopathic haemolytic anaemia. These fragmented erythrocytes may account for up to 6.9% of all circulating erythrocytes [71] and thus represent a significant cellular load that requires clearance by the reticuloendothelial system. Based upon our observations, we would speculate that IgM might play a role in the recognition and clearance of damaged erythrocytes that exhibit significant disruption of cell membrane integrity (Fig 7), though this will require further studies.

In conclusion, these flow cytometric and morphological studies indicate that circulating IgM specifically binds to cells that have progressed through apoptosis such that they have developed marked plasma membrane disruption that allows IgM to access intracellular epitopes. Studies of IgM binding to permeabilised lymphoid and parenchymal tissue cells indicate that both normal and apoptotic cells possess intracellular epitopes that can be recognised by IgM. Lastly, permeabilised erythrocytes also exhibit specific IgM binding. The totality of these data suggest that an important function of circulating IgM is to recognize and tag any nucleated circulating or resident tissue cell or erythrocytes that is severely damaged and exhibits significant plasma membrane disruption. Subsequent activation of complement and opsonisation would promote clearance of these severely damaged cells by phagocytes. Further work is required to define the role of circulating IgM in the specific clearance of nucleated cells and erythrocytes with severely compromised cell membranes *in vivo* in conditions such as ischemic injury, sepsis and thrombotic microangiopathies.

## Supporting Information

S1 Fig Non-significant IgM binding evident on mouse thymocytes following exposure to purified mouse pentameric isotype control antibodies. Mouse thymocytes were rendered apoptotic by overnight culture. Thymocytes were then exposed to either purified mouse pentameric isotype control antibodies (unconjugated mouse (Balb/c) IgMκ isotype control), PBS or Balb/c plasma (as a source of IgM). Following incubation with an anti-IgM antibody, the binding of IgM was assessed by flow cytometry. Annexin-V (AnnV) staining, assessed by flow cytometry, was used to determine the level of non-apoptotic (AnnV^-^) and apoptotic (AnnV^+^) thymocytes. **A**) Representative histogram indicating that unconjugated mouse IgM control antibodies do not bind to AnnV^+^ thymocytes. **B**) Apoptotic thymocytes were gated on the basis of AnnV positivity and the percentage of AnnV^+^IgM^+^ thymocytes was assessed. IgM binding in isotype control and PBS treatment groups remained non-significant whilst samples exposed to Balb/c plasma exhibited significant binding (one-way ANOVA; *P* < 0.0001). Data representative of *n* = 5 per group. Data expressed as means ±SEM.
(TIF)

S2 Fig IgG does not bind apoptotic mouse thymocytes. Mouse thymocytes were rendered apoptotic by overnight culture. Thymocytes were then exposed to either PBS, Balb/c plasma (as a source of IgG) or plasma from immunodeficient RAG1-deficient mice that does not contain IgG. Following incubation with an anti-IgG secondary antibody binding was assessed by flow cytometry. IgG binding in PBS, Balb/c plasma and RAG1-deficient mice plasma remained minimal. Data shown for a representative histogram (n = 4 per group).
(TIF)

S3 Fig Additional electron microscopy images of AnnV^+^IgM^-^ and AnnV^+^IgM^+^ cell populations. Mouse thymocytes rendered apoptotic by overnight culture were exposed to Balb/c plasma. On the basis of FSC/SSC thymocytes were then sorted into AnnV^-^IgM^-^, AnnV^+^IgM^-^ and AnnV^+^IgM^+^ populations (as illustrated in Fig 1D) by FACS and examined by electron microscopy. Images were taken using a Philips CM120 transmission electron microscope with a Gatan Orius CCD camera. Whole image contrast/brightness was adjusted using ImageJ. Representative images of AnnV^+^IgM^-^ and AnnV^+^IgM^+^ cells are shown.
(TIF)

S4 Fig Mouse circulating IgM binds AnnV^+^ human Jurkat cells. Human Jurkat cells were treated with camptothecin for 16-hours to induce apoptosis. Jurkat cells were then exposed to PBS or human serum as a source of IgM prior to incubation with anti-human IgM antibody. Some Jurkat cells were exposed to PBS, Balb/c plasma as a source of mouse IgM or purified mouse pentameric isotype control antibodies (unconjugated mouse (Balb/c) IgMκ isotype control). Samples were then incubated with an anti-mouse IgM antibody. IgM binding was assessed by flow cytometry. Annexin-V (AnnV) staining, assessed by flow cytometry, was used to determine the level of non-apoptotic (AnnV^-^) and apoptotic (AnnV^+^) cells. Apoptotic Jurkat cells were gated on the basis of AnnV positivity and the proportion of AnnV^+^IgM^+^ Jurkat cells was assessed. A proportion of apoptotic Jurkat cells exposed to human serum or Balb/c plasma exhibited IgM binding. Representative histograms are depicted (*n* = 4 per group).
(TIF)
