# Supplementary figures and images for: Circulating IgM Requires Plasma Membrane Disruption to Bind Apoptotic and Non-Apoptotic Nucleated Cells and Erythrocytes
Source: PLoS One. 2015 Jun 29;10(6):e0131849. doi: 10.1371/journal.pone.0131849 (PMC4488261; doi:10.1371/journal.pone.0131849)

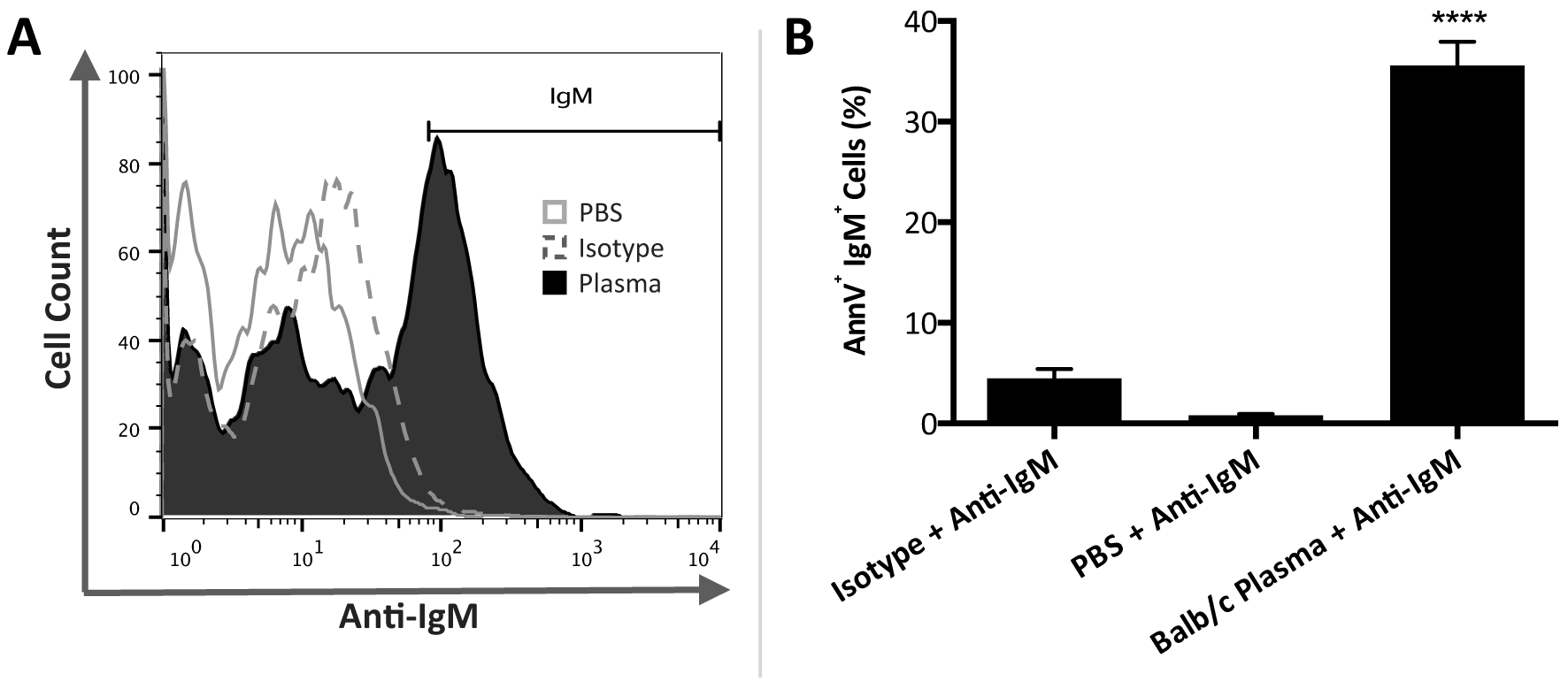

Supplement: S1 Fig — Mouse thymocytes were rendered apoptotic by overnight culture. Thymocytes were then exposed to either purified mouse pentameric isotype control antibodies (unconjugated mouse (Balb/c) IgMκ isotype control), PBS or Balb/c plasma (as a source of IgM). Following incubation with an anti-IgM antibody, the binding of IgM was assessed by flow cytometry. Annexin-V (AnnV) staining, assessed by flow cytometry, was used to determine the level of non-apoptotic (AnnV-) and apoptotic (AnnV+) thymocytes. A) Representative histogram indicating that unconjugated mouse IgM control antibodies do not bind to AnnV+ thymocytes. B) Apoptotic thymocytes were gated on the basis of AnnV positivity and the percentage of AnnV+IgM+ thymocytes was assessed. IgM binding in isotype control and PBS treatment groups remained non-significant whilst samples exposed to Balb/c plasma exhibited significant binding (one-way ANOVA; P < 0.0001). Data representative of n = 5 per group. Data expressed as means ±SEM. (TIF) [file pone.0131849.s001.tif]

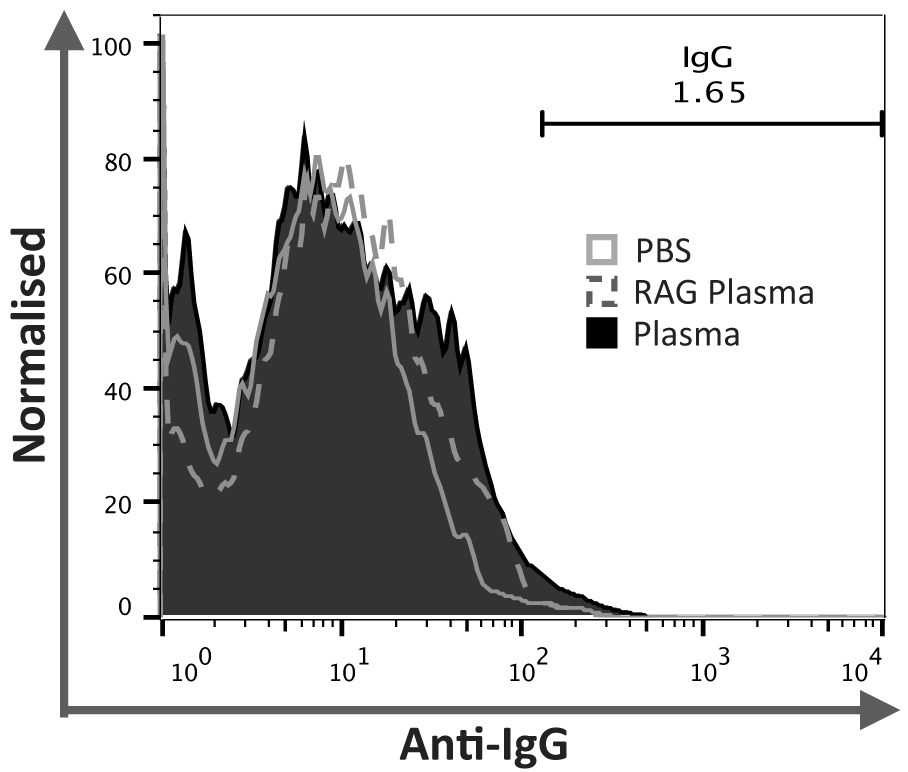

Supplement: S2 Fig — Mouse thymocytes were rendered apoptotic by overnight culture. Thymocytes were then exposed to either PBS, Balb/c plasma (as a source of IgG) or plasma from immunodeficient RAG1-deficient mice that does not contain IgG. Following incubation with an anti-IgG secondary antibody binding was assessed by flow cytometry. IgG binding in PBS, Balb/c plasma and RAG1-deficient mice plasma remained minimal. Data shown for a representative histogram (n = 4 per group). (TIF) [file pone.0131849.s002.tif]

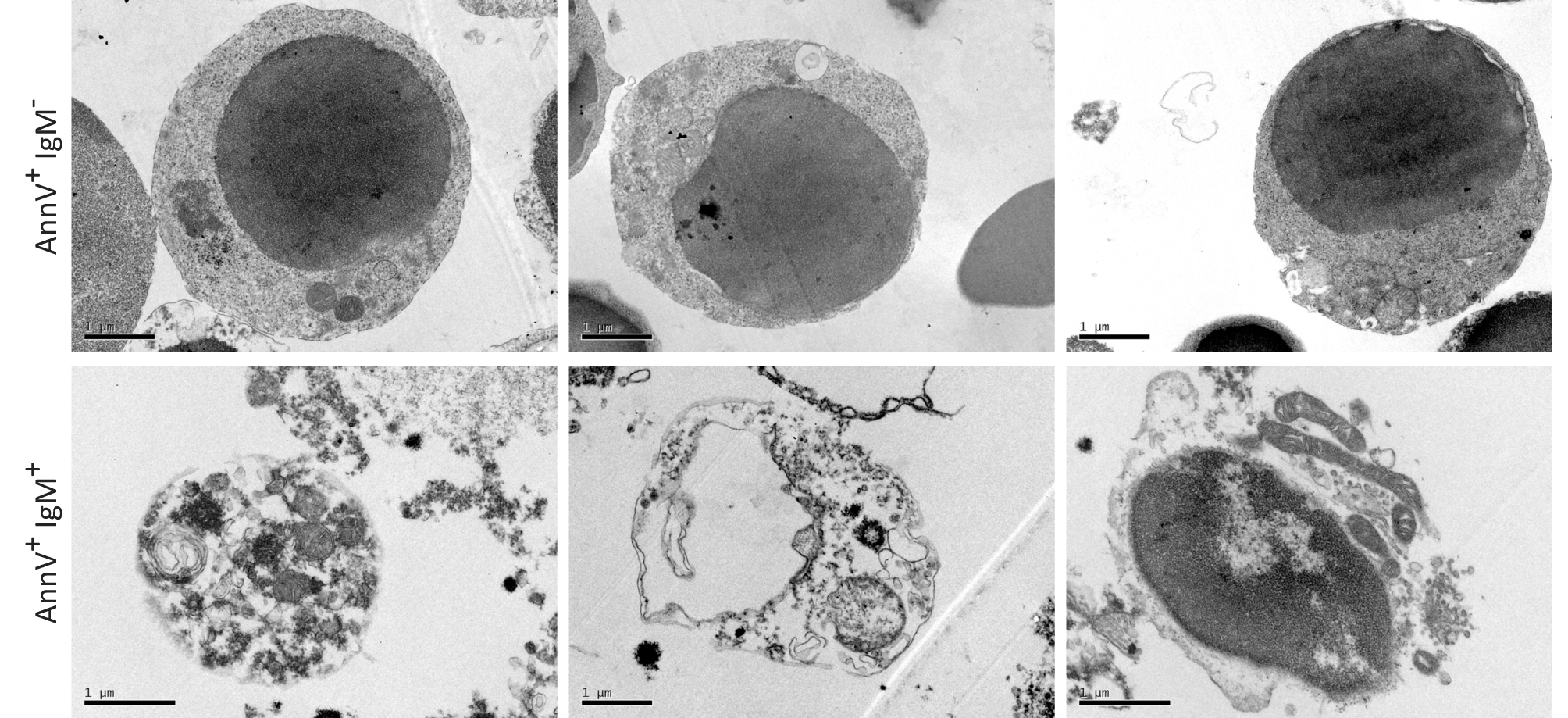

Supplement: S3 Fig — Mouse thymocytes rendered apoptotic by overnight culture were exposed to Balb/c plasma. On the basis of FSC/SSC thymocytes were then sorted into AnnV-IgM-, AnnV+IgM- and AnnV+IgM+ populations (as illustrated in Fig 1D) by FACS and examined by electron microscopy. Images were taken using a Philips CM120 transmission electron microscope with a Gatan Orius CCD camera. Whole image contrast/brightness was adjusted using ImageJ. Representative images of AnnV+IgM- and AnnV+IgM+ cells are shown. (TIF) [file pone.0131849.s003.tif]

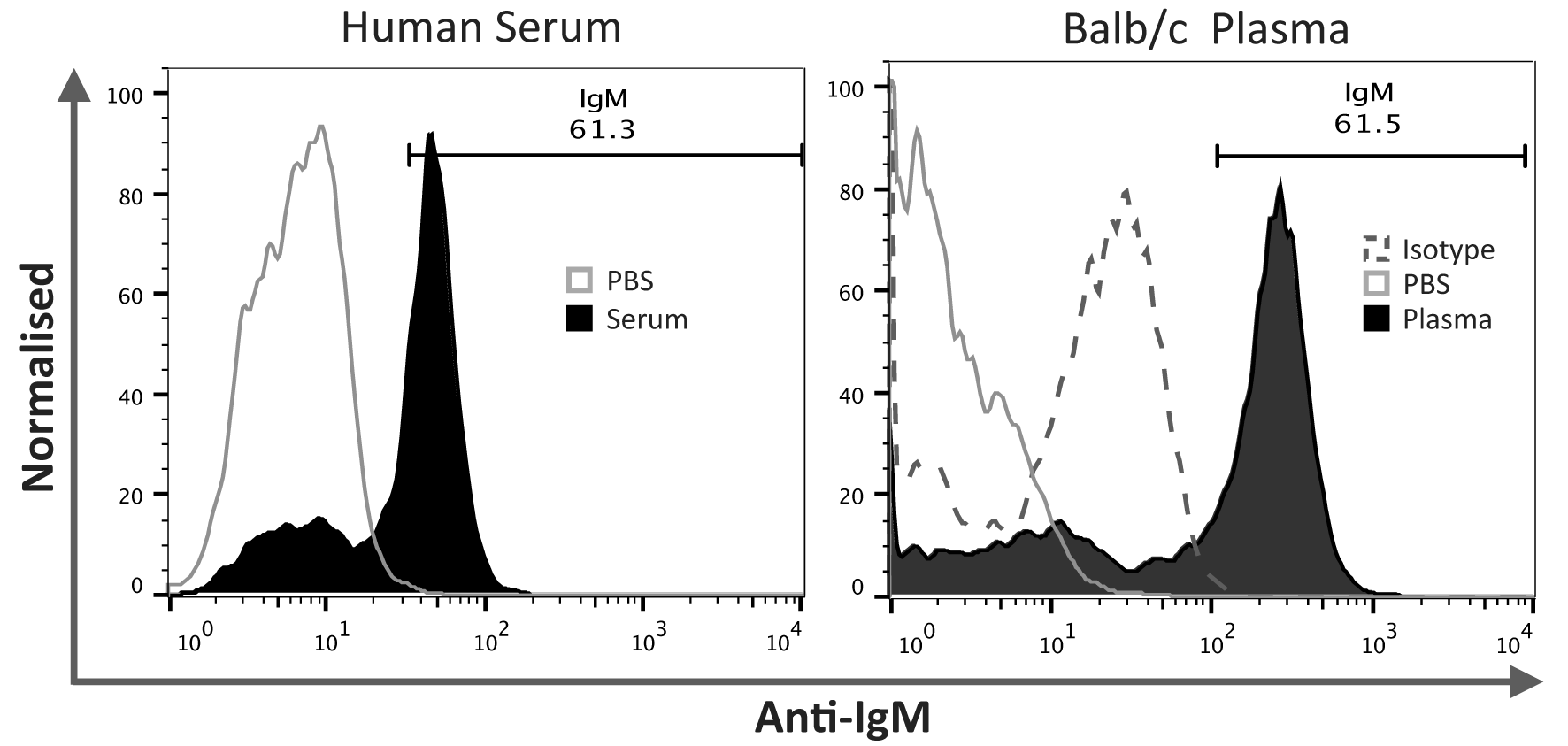

Supplement: S4 Fig — Human Jurkat cells were treated with camptothecin for 16-hours to induce apoptosis. Jurkat cells were then exposed to PBS or human serum as a source of IgM prior to incubation with anti-human IgM antibody. Some Jurkat cells were exposed to PBS, Balb/c plasma as a source of mouse IgM or purified mouse pentameric isotype control antibodies (unconjugated mouse (Balb/c) IgMκ isotype control). Samples were then incubated with an anti-mouse IgM antibody. IgM binding was assessed by flow cytometry. Annexin-V (AnnV) staining, assessed by flow cytometry, was used to determine the level of non-apoptotic (AnnV-) and apoptotic (AnnV+) cells. Apoptotic Jurkat cells were gated on the basis of AnnV positivity and the proportion of AnnV+IgM+ Jurkat cells was assessed. A proportion of apoptotic Jurkat cells exposed to human serum or Balb/c plasma exhibited IgM binding. Representative histograms are depicted (n = 4 per group). (TIF) [file pone.0131849.s004.tif]
